# Eco-efficiency and agricultural innovation systems in developing countries: Evidence from macro-level analysis

**DOI:** 10.1371/journal.pone.0214115

**Published:** 2019-04-05

**Authors:** Christian Grovermann, Tesfamicheal Wossen, Adrian Muller, Karin Nichterlein

**Affiliations:** 1 FAO—Food and Agriculture Organization of the United Nations, Rome, Italy; 2 FiBL–Research Institute of Organic Agriculture, Frick, Switzerland; 3 IITA–International Institute for Tropical Agriculture, Nairobi, Kenya; 4 ETHZ—Swiss Federal Institute of Technology Zurich, Zurich, Switzerland; University of Toronto, Rotman School, CANADA

## Abstract

Agricultural innovation is an essential component in the transition to more sustainable and resilient farming systems across the world. Innovations generally emerge from collective intelligence and action, but innovation systems are often poorly understood. This study explores the properties of innovation systems and their contribution to increased eco-efficiency in agriculture. Using aggregate data and econometric methods, the eco-efficiency of 79 countries was computed and a range of factors relating to research, extension, business and policy was examined. Despite data limitations, the analysis produced some interesting insights. For instance public research spending has a positive significant effect for emerging economies, while no statistically significant effect was found for foreign aid for research. However, foreign aid for extension is important in less developed economies. These and other results suggest the importance of context-specific interventions rather than a “*one size fits all”* approach. Overall, the analysis illustrated the potential of a macro-level diagnostic approach for assessing the role of innovation systems for sustainability in agriculture.

## 1. Introduction

High-yielding crop varieties, advanced animal breeding, mechanisation, use of agrochemicals and modern management practices have led to large increases in food production and productivity, while slowing the conversion of natural ecosystems to arable land. At the same time, intensive agricultural production puts pressures on the environment in terms of soil degradation, depletion of aquifers, biodiversity loss, nutrient pollution and pesticide contamination [1–3]. Ecology and productivity in food systems thus feature prominently in the Sustainable Development Goals.

The UN Food and Agriculture Organization, in its vision for sustainable food and agriculture, sets out clear principles for improving resource use efficiency, agro-ecosystems, livelihoods, resilience and governance [4], placing strong emphasis on the complementarities among the economic, social and environmental dimensions of sustainability. However, trade-offs across these dimensions and over time are not easy to overcome. Innovative solutions are required to maximise synergies that raise agricultural productivity, reduce food loss, improve the ways with which inputs are converted into outputs and conserve scarce resources [5]. “The present paradigm of intensive crop production cannot meet the challenges of the new millennium. In order to grow, agriculture must learn to save” [6]. This requires a focus on reducing external input use and environmental impacts of agriculture. There is thus a need to go beyond increasing technical efficiency or land, labour and total factor productivity (TFP) in agriculture, focusing rather on eco-efficiency gains. TFP, as the ratio between total outputs and total inputs, allows analysing the rate of technical change. Growth in TFP is interpreted as increased efficiency of input use [7]. However, it does not account for environmental aspects. Eco-efficiency is defined as the ratio between economic value added and a composite indicator of environmental pressures [8]. It can thus serve as a complementary efficiency or productivity measure, avoiding the shortfalls of TFP.

In addition to increased investments, streamlined policies and enhanced farming and natural resource management practices, a successful strategy for agricultural efficiency and productivity increases in developing countries involves strengthening agricultural innovation systems (AIS) [9–11]. An AIS is a network of actors (organisations and individuals) together with supporting institutions (formal and informal) and policies in the agricultural sector that bring existing or new products, processes, and forms of organisation into social and economic use [12]. Over the last 20 years, a widely recognised AIS concept has evolved [13]. Adopting an AIS perspective for agricultural development activities is gaining traction beyond the academic community with international agencies and fora, donors, governments, and research and extension organisations [9–10, 14–15]. Based on a conceptual model proposed by Arnold and Bell [16] and further refined by Spielman and Birner [17], four main domains characterise an AIS: (1) Research and education, involving private and public research institutes, universities and vocational training centres; (2) Business and enterprise, involving various value chain actors, agribusiness, producers and consumers; (3) Bridging institutions, involving stakeholder platforms, contractual arrangements and various types of rural advisory services; (4) Enabling environment, involving governance and policies as well as behaviours, mind-sets and attitudes. Moving towards sustainable growth in the food and agriculture sectors needs a strong evidence base on what works and what does not [5]. However, the complex nature of the AIS concept and innovation processes poses challenges for analytical work in terms of data availability and methodology.

The importance of AIS for efficiency and productivity gains motivates this investigation into whether AIS may also play an important role in strategies for increasing eco-efficiency. From the literature, there is so far little evidence on whether and what type of AIS properties can contribute to increased eco-efficiency. This paper addresses this gap by econometrically estimating the influence of various AIS characteristics on eco-efficiency in agriculture for a sample of low- and middle-income developing and transition countries.

The literature on innovation systems in agriculture largely focuses on descriptive methods and avoids the use of formal models (e.g. [18–21]). The same applies to studies taking an innovation systems perspective towards sustainability issues (e.g. [22–24]). Besides combining eco-efficiency and AIS analysis for the first time, we also add to previous assessments in methodological terms by using an econometric model to explore the question of how innovation systems can contribute to increased eco-efficiency. For this, we adopt and extend the approach proposed by Mekonnen et al. [11].

The next section gives an overview of the key concepts used in the analysis. Subsequently, the data, and methods are explained (sections 3 and 4). Section 5 presents the efficiency scores by country and shows the determinants of technical and eco-efficiency. In section 6, the findings are then further scrutinised and discussed in terms of their relevance for policy-making, while also pointing out some limitations of the analysis.

## 2. Conceptual frame

While eco-efficiency clearly relates to sustainability, improvements in eco-efficiency do not guarantee sustainability [25]. Pollution levels might still be beyond the carrying capacity of the agro-ecosystems. Nevertheless, their interpretation in relative terms allows for comparison of performance across time and space. It must also be underlined that measures used for eco-efficiency analysis do not attempt to represent environmental impact of agricultural production in a given country, but rather the environmental pressures associated with it. Following the eco-efficiency definition provided by Kuosmanen and Kortelainen [8], a country in our study is considered eco-efficient if it is impossible to decrease any environmental pressure without simultaneously increasing another pressure or decreasing the economic value added.

Data Envelopment Analysis (DEA) is widely used for eco-efficiency analysis and has been applied to assess eco-efficiency at the farm level [26–27]. It is a non-parametric approach that uses linear programming techniques to envelop observed input–output vectors for estimating the underlying frontier and efficiency (distance from the frontier). We use DEA in order to obtain eco-efficiency scores for agriculture across low and middle-income countries. Subsequently, we analyse the relationship between innovation system properties and eco-efficiency. While the properties are expected to have a positive effect on technical efficiency, their impact on eco-efficiency is less clear. Therefore, this exploratory analysis aims at shedding light on the characteristics of an AIS that can contribute to or may rather hinder a transition towards more eco-efficient production. By computing technical efficiency scores to complement the assessment of eco-efficiency, it is then possible to distinguish key differences in what drives either type of efficiency.

More precisely, the overall approach taken in this study can be described as Latent Class Data Envelopment Analysis (LCDEA). We enhance the standard DEA method because countries in our dataset are heterogeneous in terms of technological choice and AIS characteristics. It cannot necessarily be assumed that all Decision-Making Units (DMUs) operate under similar technological circumstances and share a single efficiency frontier. For example, Brazil and China tend to use more machinery, capital and fertiliser compared with countries in sub-Saharan Africa, such as Rwanda and Uganda. The use of a latent class model allows us to focus on within class differences by estimating class-specific eco-efficiency and technical efficiency scores. Thereby countries are classified in terms of technology choice rather than geographic location or any other *a priori* criteria unrelated to the analysis. Such arbitrary categorisation has been widely criticised (e.g. [8, 26–28]) because it fails to capture adequately within and between region differences in technology choice and use [11]. Through the LCDEA approach groups can be created that are more homogeneous in the level and type of technology use. Such classification is appropriate because production technologies and environmental pressures are closely related. Comparing results between groups is valuable for identifying relevant policy implications.

## 3. Data

The availability of comprehensive aggregate data posed a substantial challenge for this type of cross-country analysis. A range of data sources was used to create a dataset with the necessary information on environmental pressures, agricultural outputs and inputs, as well as AIS indicators. With the gathered data 79 low- and middle-income countries and a period ranging from 2004 to 2011 could be covered. For more recent years insufficient data was available. For inputs, outputs and AIS properties, we relied on data very similar to those used by Mekonnen et al. [11]. The innovation system characteristics were complemented with additional variables, for instance on research spending in agriculture or foreign aid for agricultural extension. The choice of variables representing AIS characteristics was discussed with subject matter experts and deemed adequate, considering the limited availability of data that is more agriculture-specific. Regarding environmental pressures, we required complete time-series information for eight years, which can be meaningfully used at an aggregate level of analysis. This implies that variables should reflect national pollution levels rather than just average values of point-specific pollution. The analysis focuses on developing countries in Africa (28), Asia (16), Eastern Europe (16) and Latin America (19), where the need for intensification is considerable, while agro-ecosystems are increasingly under pressure. Based on the number of countries and the specified time range, with few missing values, we obtained a large dataset of 608 observations for conducting the analysis.

Table 1 gives an overview of the variables used in the eco-efficiency and technical efficiency analyses respectively. Values on emissions from agriculture were obtained from the Climate Analysis Indicators Tool [29]. This variable serves as a proxy for the pressures of intensive agricultural production with high external input levels. Environmental contamination by pesticides was measured through the pesticide regulation score, which is part of the Environmental Performance Index [30]. This score quantifies whether countries allow, restrict or ban the ‘Dirty Dozen’ Persistent Organic Pollutants (POPs) under the Stockholm Convention. Fertiliser use and land under irrigation were included in the eco-efficiency DEA model as proxies for nutrient pollution and water withdrawal by agriculture. They are also part of the technical efficiency analysis, along with labour, land, machinery and annual rainfall variables. For most of the countries considered in the analysis, rainfall strongly affects harvest as production is predominantly rainfed. It is thus a key input for production. The information on productivity enhancing inputs as well as economic value added was compiled by Fuglie [7] for TFP analysis. This dataset, available in its most recent version from USDA [31], is primarily based on annual time series information from FAOSTAT and, in some cases, modified or supplemented with data from other sources (such as national statistical agencies), when they were considered to be more accurate or up-to-date.

**Table 1:** Summary statistics of variables used in the efficiency analysis.

Efficiency analysis variables	Mean	St. Dev.	Min	Max
*Output*				
Value of agricultural output (1,000 int. dollars)	17,600	57,000	47.2	5,120,000
*Eco-efficiency*				
Total GHG emissions from agriculture (MtCO2e/ha)	323	1070	0.21	10,596
Pesticide regulation score (0 to 25)	16.93	7.2	0	24
*Both eco-efficiency and technical efficiency*				
Fertiliser (tonnes of nutrients)	1,561	6,669	0.001	63,600
Land under irrigation (1,000 ha)	2,882	10,341	0.8	66750
*Technical efficiency*				
Labour (1,000 people)	14,790	62,934	27	506031
Land (1,000 ha)	11,464	26,363	47	159450
Value of machinery used in agriculture (1,000 int. dollars)	257.7	956.8	0.04	10066.3
Annual rainfall (mm)	1171	837	28	3676
Observations (#)	608			

Table 2 summarises the information on AIS characteristics used as determinants of eco-efficiency in the regression analysis. The choice of variables is motivated by the AIS concept as specified in key reports on the topic [9, 12, 17]. Each variable is attributed to one of the four AIS domains, capturing education and research levels, bridging institutions, business and enterprise development and enabling environment aspects. The variables thus measure the innovation system characteristics of the respective countries. As some variables could capture properties of two domains, we consider them as such, representing them by the overlapping lines in Table 2. Mekonnen et al. [11] pointed out that the AIS variables are expected to have a positive influence on the technical efficiency of agricultural production. For instance, business and enterprise indicators are expected to affect it through their influence on the nature and performance of business and business innovation in the agricultural sector. The quality of institutions and legal systems is assumed to enable innovation in agriculture. Through our study we contend that all the positive relationships between innovation system characteristics and technical efficiency postulated by Mekonnen et al. [11] also apply in the case of eco-efficiency.

**Table 2:** Summary statistics of AIS characteristics and their link to the AIS domains.

AIS domains	Explanatory AIS variables	Mean	St. Dev.	Min	Max
Education & research	Quality of the educational system (1 = low to 7 = high)	3.30	0.68	1.91	5.30
	Primary school enrolment (gross)	106.00	13.51	51.00	164.50
	Agricultural researchers (FTEs per 100,000 farmers)	912.1	1,007.20	35.00	7520.60
	Agricultural research spending (% of agr. GDP)	0.82	0.89	0.11	7.42
	Foreign aid for agricultural research (% of agr. GDP)	0.013	0.04	0.00	0.37
	Scientific and technical journal articles (#)	1969	7445	1.00	74,019.00
Bridging institutions	University-industry collaboration in R&D (1 = minimal to 7 = intensive)	3.10	0.64	1.60	4.98
	Foreign aid for extension (% of agr. GDP)	0.02	0.05	0.00	0.45
	Mobile cellular subscriptions (# per 100 people)	62.13	39.30	0.21	189.00
Business & enterprise	Start-up procedures to register a business (#)	9.17	3.22	2.00	18.00
	Time required to start a business (days)	37.0	29.8	2.00	153.00
	Total tax rate (% of commercial profits)	51.45	39.9	14.4	292.40
	Ease of accessing loans (1 = low to 7 = high)	2.80	0.66	1.38	4.65
Enabling environment	Credit information index (0 = low to 8 = high)	3.35	2.10	0.00	6.00
	Agricultural policy costs (1 = low to 7 = high)	3.8	0.57	2.16	5.50
	Legal rights index (0 = weak to 12 = strong)	4.96	2.18	0.00	10.00
	Foreign aid received (current int. US$ per capita)	52.20	57.40	0.07	672.50
	Gross capital formation (% of GDP)	24.70	7.20	3.03	62.50
	Health expenditures (% of GDP)	6.20	1.81	2.40	12.80

Data on the quality of the educational system, on R&D collaborations between university and industry, on the ease of accessing loans, as well as on agricultural policy costs, are available through the Global Competitiveness Report published by the World Economic Forum [32]. The variables rank countries on a scale from 1 (low/minimal) to 7 (high/intensive). The Agricultural Science and Technology Indicators (ASTI) database provides information on national agricultural research spending and workforce [33], while we rely on data collected by the Development Assistance Committee of the Organisation of Economic Cooperation and Development (OECD) for foreign aid statistics related to agricultural research and extension [34]. The majority of the figures characterising the properties of a country’s innovation system are taken from the World Bank’s World Development Indicators [35]: Rate of primary school enrolment, the number of scientific and journal articles, mobile phone subscriptions, start-up procedures and time to register a business, total tax rate, domestic credit to private sectors, credit information index, legal rights index, foreign aid received, gross capital formation and health expenditures.

## 4. Methodology

Herein, we present how we combined latent class analysis with DEA to examine the eco-efficiency and technical efficiency scores. In developing the LCDEA model, we followed two steps to obtain class-specific eco-efficiency and technical efficiency scores. First, we ran a latent class model using technology choice variables to determine class membership. We then performed a DEA to determine class-specific eco-efficiency and technical efficiency scores.

### 4.1 Latent class model

Consider a general production function expressed as a function of conventional agricultural input variables (e.g., land) and environmental pressure variables (e.g., pesticide regulation score) that influence technological choice and are thus summarized under the term “technological choice variables”:

$$Y_{it}=\sum_{k=1}^{K}(\alpha_{k}+\gamma_{k}x_{kit})+\varepsilon_{it|j}$$ (1)

where *Y*_*it*_ is the value of agricultural output for country *i* at time *t*. *x*_*kit*_ is the vector technological choice variables *k* = 1,…,*K*. The latent class model assumes that there are *j* distinct classes for parameters *α* = α_1_,α_2_,α_3_…α_*K*_ and *γ* = γ_1_,γ_2_,γ_3_…γ_*K*_ that define countries based on their technological choice (subscript *t* is subsequently dropped for simplification). Class membership status *j* of the countries is unknown *a priori* and depends on their technological choice. Following Llorca et al. [36], let all the parameters associated with class *j* be denoted by *θ*_*j*_. The conditional likelihood function of country *i* belonging to class *j* is then denoted by LF_*ij*_(*θ*_*ij*_). The unconditional likelihood function is then computed as a weighted sum of the likelihood function across the *j* classes, where the weights are the probabilities of class membership *π*_*ij*_(*δ*_*j*_):

$${\mathrm{LF}}_{i}\left(\theta ,\delta \right)=\overset{J}{\underset{j=1}{\sum }}{\mathrm{LF}}_{ij}(\theta_{j})\pi_{ij}\left(\delta_{j}\right)$$ (2)

with ${0\leq \pi }_{ij}(\delta_{j})\leq 1\;and\;\sum_{j=1}^{J}\pi_{ij}(\delta_{j})=1.$

The above condition is satisfied by parameterising class probabilities as a multinomial logit model in the following fashion:

$$\pi_{ij}(\delta_{j})=\frac{\exp\left(\delta_{j}^{\prime }x_{i}\right)}{\sum_{j=1}^{J}\exp\left(\delta_{j}^{\prime }x_{i}\right)}$$ (3)

Where *x*_*i*_ is the vector of technological choice variables that determine class membership and $\delta_{j}^{\prime }$ are the corresponding class-specific parameters to be estimated. In our setting, we used the following variables to determine class membership: fertiliser use, arable land, land under irrigation, rainfall, machinery, labour and pesticide regulation score. In addition to the above inputs, we also used total emissions from agriculture, which is an outcome of technology choice rather than a variable that affects technological choices, in the class splitting model. We introduced this variable as a proxy for the type of technology used by countries. We assume that differences in these environmental pressure and conventional inputs between countries affect their technological choice and level of eco-efficiency. Therefore, countries that possess similar attributes in the above variables are more likely to be in the same class. The overall likelihood function is then derived based on Eq (2) and Eq (3) as follows:

$$lnLF(\theta ,\delta )=\sum_{i=1}^{I}{lnLF}_{i}(\theta ,\delta )=\sum_{i=1}^{I}ln\{\sum_{j=1}^{J}{LF}_{ij}(\theta_{j})\pi_{ij}(\delta_{j})\}$$ (4)

The parameters of the log-likelihood function in Eq (4) can be estimated using maximum likelihood. The posterior class membership probabilities are then computed as follows:

$$\pi (j|i)=\frac{{LF}_{ij}({\hat{\theta }}_{j})\pi_{ij}({\hat{\delta }}_{j})}{\sum_{j=1}^{J}{LF}_{ij}({\hat{\theta }}_{j})\pi_{ij}({\hat{\delta }}_{j})}$$ (5)

By applying the Schwarz Bayesian Information Criterion (SBIC), we can determine the optimal class size. Once the number of classes are determined, posterior probabilities from Eq (5) can be used to assign each country to their respective class based on their highest posterior probability.

### 4.2 DEA for determining eco-efficiency and technical efficiency

After determining the number of latent classes, we used DEA to estimate class-specific eco-efficiency and technical efficiency scores. We employed DEA and not a stochastic frontier production approach as it allows multiple inputs–outputs relationships without any assumption on the underlying functional relationship linking inputs, environmental pressures and outputs [8, 26]. Following Kuosmanen and Kortelainen [8] and Picazo-Tadeo et al. [26], we assume that a given country *i* produces a total output, which is captured as the value of agricultural output *Y*_*i*_, using inputs that may have a detrimental effect on the environment denoted by *D*_*n*_ (*n* = 1,2,3,……*N*). Given our definition of eco-efficiency (*EF*) as the ratio of economic value added to environmental damage, it is formalised as follows:

$${EF}_{i}=\frac{Y_{i}}{{f(D}_{i1},\ldots ,D_{iN})}$$ (6)

Where *f*(.) is the damage function that aggregates individual *N* environmental pressures into a single environmental damage score [8]. As pointed out by Kuosmanen and Kortelainen [8] and Picazo-Tadeo et al. [26], constructing the composite environmental pressure score that aggregates individual environmental pressures is not straightforward, as it requires a weighting mechanism that takes into account the relative importance of the different environmental pressures. There are many approaches to calculating weights. These include assigning the same weight to each individual environmental pressure variable or assuming any arbitrary linear combination of the environmental pressure variables: ${f(D}_{i1},\ldots ,D_{iN})=\sum_{n=1}^{N}\omega_{n}D_{in}$, where *ω*_*i*_ is a vector of weights. A natural way of assigning weights in the latter case would be to use Principal Component Analysis (PCA). However, PCA relies on orthogonal transformations of the environmental pressure variables. DEA overcomes the problem of choosing weights for the linear combination without requiring an arbitrary combination. It maximizes the relative eco-efficiency score of a given DMU compared with the other DMUs (in our case it maximizes the relative eco-efficiency score compared to the class specific scores) by accounting for all environmental pressure variables. Formally, the DEA eco-efficiency score can be calculated as

$${Maximize}_{\omega_{ni}}{EF}_{i}=\frac{Y_{i}}{\sum_{n=1}^{N}\omega_{in}D_{in}}$$ (7)

s. t.

$$\frac{Y_{i}}{\sum_{n=1}^{N}\omega_{in}D_{in}}\leq 1\;\mathrm{and}\;\omega_{in}\geq 0$$ (8)

Following Picazo-Tadeo et al. [26], the dual formation of the above maximization problem can be formalized as follows:

$${Minimize}_{{\tau_{i,}\varphi }_{i^{\prime }}}\;{EF}_{i^{\prime }}=\varphi_{i^{\prime }}$$ (9)

s. t.

$$Y_{i^{\prime }}\leq \sum_{i=1}^{I}\tau_{i}Y_{i}\;\mathrm{and}\;\varphi_{i^{\prime }}D_{ni^{\prime }}\geq \sum_{i=1}^{I}\tau_{i}D_{in}\;\mathrm{and}\;\tau_{i}\geq 0$$ (10)

In the above dual formalization, the strictly non-negative (*τ*_*i*_) denotes the weighting of each country in the composition of the eco-efficient frontier. $\varphi_{i^{\prime }}^{*}$ (which is the optimal solution of the above minimization problem) denotes country specific eco-efficient scores. A higher eco-efficiency score suggests that reducing environmental pressures is more difficult without reducing output.

DEA takes into account heterogeneities within the observed sample and uses the best performing unit as a benchmark to which other units in the sample are compared. Therefore, the eco-efficiency and technical efficiency scores of countries as obtained from DEA are measured relative to the “best practice” in the sample, which is not necessarily the same as the “best available” technology [11]. It is also important to note that the analysis is based on a latent class model, so eco-efficiency and technical efficiency scores of countries are class specific and calculated relative to the best practice in the class to which the sample unit belongs. Under this circumstance, eco-efficiency and technical efficiency scores cannot be directly compared between classes. Once the class-specific eco-efficiency and technical efficiency scores were calculated, we analysed the determinants of efficiency (both eco-efficiency and technical-efficiency) using the bootstrapped truncated regression approach. This approach proposed by Simar and Wilson [37] is superior to traditional approaches (Tobit and OLS). Simar and Wilson [37] showed that regressing DEA efficiency scores on socio-economic characteristics using a Tobit model has several limitations: These include the fact that DEA efficiency scores are bounded between zero and one but not within the context of a censoring data generating process. In addition, efficiency estimates from DEA are also serially correlated, leading to invalid inference [37]. We therefore employed bootstrapped truncated regression to examine the determinants of both eco-efficiency and technical-efficiency.

## 5. Results

In this section, we first explain the efficiency scores computed for each country and then present the determinants of eco-efficiency and technical efficiency.

### 5.1 Class specific eco-efficiency scores

**Fig 1:**
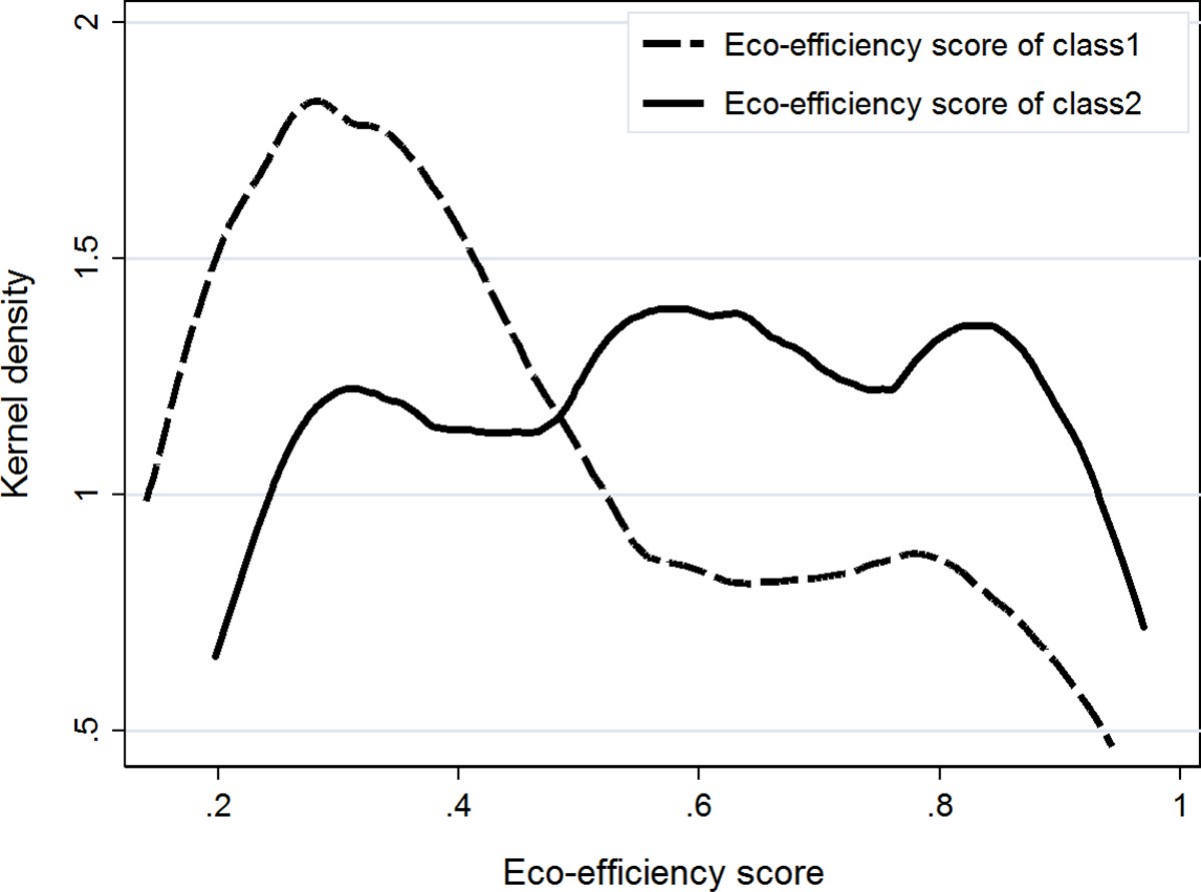
The distribution of class-specific eco-efficiency scores.

We provide eco-efficiency scores calculated using the class splitting model. Based on the SBIC we identified the optimal number of latent classes for estimating efficiency scores, which resulted in a two-class model for our specification. Fig 1 reports the distribution of eco-efficiency scores for the two classes. A country will be considered as eco-efficient if the DEA eco-efficiency score is one. If the score is less than one, then it will imply inefficiency suggesting a reduction in environmental pressure variables is feasible without reducing agricultural output. The distribution of the class-specific eco-efficiency scores suggest a clear difference between countries in terms of eco-efficiency. In terms of the composition of countries, a distinct grouping was established. Class one predominantly consists of emerging economies with a generally more commercial agricultural sector. For instance, Brazil, China, India and South Africa are members of class one. The average eco-efficiency score of countries in this class (being 0.41) is low and remained constant between 2004 and 2011. Class two is composed mostly of developing countries, but also includes several smaller emerging economies. While the trend is similar for class two countries, the average eco-efficiency scores remained at a higher level (being 0.59). Despite differences, the results suggest for both classes that most countries can reduce environmental pressures without reducing the value of agricultural output. Note that eco-efficiency scores are class specific and not comparable across classes. On average, the most eco-efficient country in class one is Rwanda, but also China and Brazil are performing relatively well. Indonesia turned out to be the most eco-efficient country in class two, followed by Costa Rica and Argentina. When comparing the most and least eco-efficient countries in terms of environmental pressures and use of conventional inputs, the result suggests that less eco-efficient countries have a lower pesticide regulation score, use more fertiliser and have higher emission levels.

### 5.2 Determinants of eco-efficiency

Table 3 shows the results of regressing AIS properties on eco-efficiency for both classes. The coefficient for scientific publishing, as a bridge between research and other domains, is positive and significant across classes. Similar trends can be observed for the quality of the educational system, and the legal right index. Overall, the results suggest that improving scientific output, education and legal rights plays an important role for eco-efficiency strategies. However, for primary school enrolment, the effect is only positive for class one countries, while it is negative for class two countries. Albeit the magnitude of this effect is small. The negative result in class two countries is likely due to the fact that a high ratio for this indicator in developing countries reflects a substantial number of overage children enrolled rather than a successful education system. Also for other AIS properties we found clear heterogeneous effects. For example, further sign reversal effects can be observed for agricultural research spending. The sign here is negative in case of class 2, while its effect is positive for class 1 countries. This can be an indication of research investments in developing countries involving limited compliance with environmental safeguard standards. Finally, health expenditures show a heterogeneous effect as well. We presume that greater spending for health care in order to compensate for higher pollution levels is associated with more intensive commercial agriculture and thus lower eco-efficiency in emerging economies. This is not the case for class 2 countries, where higher expenditures for health appear to be linked with greater eco-efficiency. One possible explanation is that agricultural activities are relatively more labour intensive in this class, so health care spending can improve the supply of farm labour, which is then available for soil conservation or integrated pest management. Furthermore, foreign aid for extension, agricultural policy costs and credit information index were positive and significant for countries in class two only.

**Table 3:** Determinants of eco-efficiency.

	Class 1	Class 2
	Coef.	Coef.
Quality of the educational system (1 = low to 7 = high)	0.114***	0.185***
	(0.018)	(0.022)
Primary school enrolment (% gross)	0.002**	-0.002**
	(0.001)	(0.001)
Agricultural researchers (FTEs per 100,000 farmers)	-0.001***	-0.001*
	(0.000)	(0.000)
Agricultural research spending (% of agr. GDP)	0.034***	-0.064***
	(0.011)	(0.021)
Foreign aid for agricultural research (% of agr. GDP)	-0.005	-0.416
	(0.282)	(0.303)
Scientific and technical journal articles (#)	0.053***	0.025***
	(0.007)	(0.008)
University-industry collaboration in R&D	-0.089***	-0.104***
(1 = minimal to 7 = intensive)	(0.024)	(0.024)
Foreign aid for extension (% of agr. GDP)	0.545	1.481***
	(0.366)	(0.448)
Mobile cellular subscriptions (# per 100 people)	-0.000	-0.000
	(0.000)	(0.000)
Start-up procedures to register a business (#)	0.005	0.001
	(0.004)	(0.005)
Time required to start a business (days)	-0.000	0.001
	(0.000)	(0.000)
Total tax rate (% of commercial profits)	-0.000*	-0.001
	(0.000)	(0.001)
Ease of accessing loans (1 = low to 7 = high)	-0.065***	-0.087***
	(0.020)	(0.019)
Credit information index (0 = low to 8 = high)	0.007	0.025***
	(0.006)	(0.006)
Agricultural policy costs (1 = low to 7 = high)	0.008	0.060**
	(0.020)	(0.023)
Legal rights index (0 = weak to 12 = strong)	0.012*	0.018***
	(0.006)	(0.006)
Foreign aid received (current int. US$ per capita)	0.000	-0.000
	(0.000)	(0.000)
Gross capital formation (% of GDP)	0.001	-0.002
	(0.001)	(0.002)
Health expenditure (% of GDP)	-0.017***	0.024***
	(0.006)	(0.008)
Number of bootstr. Reps	100	100
Wald chi2(27)	560.6 ***	371.5 ***
Prob > Chi2(27)	0.0000	0.0000
N	245	363

Bootstrapped standard errors are reported in parenthesis.

***, ** and * refers to significant at 1%, 5% and 10% respectively. Time dummies are included but not reported here

The variables defined as ease of accessing loans, number of agricultural researchers and university-research collaboration in R&D were associated negatively with eco-efficiency for both classes. Easy access to loans can create incentives for intensification investments that prioritise productivity gains over environmental concerns. The same prioritisation might be made by agricultural researchers. The effect of university-industry collaboration in R&D suggests that currently innovations resulting from such collaborations are not geared towards eco-efficiency. As reported in Table 4, this variable has a statistically significant positive effect on technical efficiency scores in class 2. Future collaboration in R&D, e.g. in the area of climate smart agriculture, will therefore matter for improving the eco-efficiency of countries, while maintaining higher levels of technical efficiency.

**Table 4:** Determinants of technical efficiency.

	Class 1	Class 2
	Coef.	Coef.
Quality of the educational system (1 = low to 7 = high)	0.008	0.017**
	(0.008)	(0.007)
Primary school enrolment (% gross)	0.000	-0.001**
	(0.000)	(0.000)
Agricultural researchers (FTEs per 100,000 farmers)	-0.000	0.000
	(0.000)	(0.000)
Agricultural research spending (% of agr. GDP)	0.002	-0.003
	(0.004)	(0.007)
Foreign aid for agricultural research (% of agr. GDP)	0.052	0.010
	(0.103)	(0.115)
Scientific and technical journal articles (#)	0.008**	-0.001
	(0.003)	(0.003)
University-industry collaboration in R&D	0.007	0.027***
(1 = minimal to 7 = intensive)	(0.009)	(0.008)
Foreign aid for extension (% of agr. GDP)	0.137	0.237*
	(0.120)	(0.136)
Mobile cellular subscriptions (# per 100 people)	0.000*	-0.000*
	(0.000)	(0.000)
Start-up procedures to register a business (#)	0.004**	0.004**
	(0.002)	(0.002)
Time required to start a business (days)	-0.000	-0.000
	(0.000)	(0.000)
Total tax rate (% of commercial profits)	0.000**	0.000
	(0.000)	(0.000)
Ease of accessing loans (1 = low to 7 = high)	0.013*	0.000
	(0.007)	(0.007)
Credit information index (0 = low to 8 = high)	-0.004	0.008***
	(0.003)	(0.002)
Agricultural policy costs (1 = low to 7 = high)	0.002	-0.018**
	(0.007)	(0.008)
Legal rights index (0 = weak to 12 = strong)	0.004	-0.003
	(0.003)	(0.002)
Foreign aid received (current int. US$ per capita)	-0.000	-0.000
	(0.000)	(0.000)
Gross capital formation (% of GDP)	-0.001**	0.001*
	(0.001)	(0.001)
Health expenditure (% of GDP)	0.000	-0.001
	(0.002)	(0.002)
Number of bootstr. reps	100	100
Wald chi2(27)	148.6***	73***
Prob > Chi2(27)	0.0000	0.0000
N	245	363

Bootstrapped standard errors are reported in parenthesis.

***, ** and * refers to significant at 1%, 5% and 10% respectively.

Time dummies are included but not reported here

### 5.3 Determinants of technical efficiency

Since the magnitude of technical efficiency does not have much policy implication by itself, we present and examine the main determinants of technical efficiency. As explained in the introduction, the focus of this paper is not on technical efficiency. Nonetheless, we report technical efficiency regression results to explore differences in the magnitude and direction of expected effects of AIS characteristics on technical and eco-efficiency scores. Certain variables that may improve technical efficiency may not necessarily improve eco-efficiency.

The average technical efficiency score for both classes is about 0.92. Despite significant differences in eco-efficiency scores between the two classes, we did not find any variation in the level of technical efficiencies. This indicates that high technical efficiency scores are not necessarily accompanied by higher eco-efficiency scores. Understanding the expected effects of AIS variables on technical and eco-efficiency respectively will be useful when considering policy objectives. Class-specific technical efficiency scores are not reported here due to space limitation but the distribution of these values is presented in the appendix.

Regarding the respective determinants of technical and eco-efficiency, similarities and differences emerge. The results in Table 4 demonstrate that the quality of the educational system, university-industry collaboration in R&D, number of start-up procedures, credit information index and total tax rate are associated with technical efficiency. The variable representing agricultural research spending was positively associated with eco-efficiency, while its effect on technical efficiency was insignificant. Foreign aid for extension appears to be of relevance for both types of efficiency for countries in class two. However, university-industry collaborations and the number of start-up procedures are positively associated with technical efficiency, but not with eco-efficiency.

## 6. Discussion and conclusions

To date cross-country studies on R&D and AIS have focused on investigating effects on agricultural productivity and technical efficiency. However, little evidence exists on which innovation system properties can support a country’s process of sustainable intensification through enhancing eco-efficiency. In the light of the Sustainable Development Goals and the multiple challenges of hunger eradication, poverty reduction, better nutrition and healthier ecosystems, metrics for better understanding policy-relevant issues related to agriculture and the environment need to be explored more widely and deeply. Eco-efficiency can capture potential trade-offs as well as synergies. It not only takes into account relations between the economic and environmental dimensions, but also the risk of shifting environmental impacts from one area to another. This safeguards against reaching potentially false conclusions when using single metrics, such as carbon footprint or pesticide contamination scores [38]. Neither could a composite one-dimensional sustainable agriculture index capture trade-offs.

Eco-efficiency analysis can offer clues on management and decision-making parameters, especially by identifying drivers in a given context, as shown in this study. Research, extension, business and policy-making are key factors in the intensification and commercialisation of farming systems around the world and their role needs to be better understood. Contrary to the great majority of AIS studies, analysing case-specific innovation processes [19, 24], this study uses aggregate data and econometric methods to explore the extent to which innovation system properties relate to eco-efficiency. Data availability poses a challenge though and little evidence from the literature exists for corroborating results found here. Therefore, at this stage, our enquiry remains exploratory rather than allowing for reliable predictions of what system properties determine eco-efficiency in agriculture.

Besides limited availability of time-series data on environmental pressures, the representation of AIS properties constitutes an important constraint in the present analysis. Due to a lack of more specific data at such an aggregate level of analysis on aspects related to e.g. quality of agricultural education and training, public spending on extension services, responsiveness of research to needs of producers or costs of certification procedures in agriculture, many of the variables in the analysis are broad and rather serve as proxies. With efforts to collect more detailed data for the sector through the Enabling the Business of Agriculture indicators [39], the precision in capturing some important elements of a country’s AIS will improve, in particular with regard to the business and enterprise domain. However, there is a need to fill data gaps related to research, education and extension, in particular with regard to depicting AIS qualities. The ASTI database records numbers of researchers and public spending on research in agriculture, but falls short of providing any indicators on the relevance and demand-orientation of agricultural research [33]. A lack of structured country data is particularly apparent for extension and other institutional arrangements that fulfil the bridging function between education and research actors on the one side and value chains actors on the other.

Despite limitations arising from the nature of the data used, the analysis leads to important insights. Eco-efficiency scores among the countries considered in this study are relatively low for both classes, while technical efficiency scores are generally high. This suggests that eco-efficiency could be improved for many countries under current conditions. Through the right organisational, institutional, social and financial combinations, existing innovations can be brought into greater use. The AIS indicators explored in this study represent potential parameters to boost innovation processes in support of eco-efficiency. Involving key national and international stakeholders and mainstreaming eco-efficiency criteria within existing development strategies will accelerate the transformation towards more sustainable and resilient rural societies.

While there is little congruence in terms of the influence of factors introduced in the study of Mekonnen et al. [11] and that of the eco-efficiency determinants, few variables have a similar effect, such as the quality of the educational system and legal right index. Critically, foreign aid for extension can boost both efficiency types, at least for countries in class two. Investing in education and extension services could thus contribute to improving technical efficiency as well as eco-efficiency. On the contrary, collaboration between universities and industry in R&D, had a positive effect on technical efficiency, but a negative effect on eco-efficiency. Possibilities to adjust modalities of collaboration might need to be considered in such instances.

This study underscores that cross-country comparison of eco-efficiency needs to take into account variation among countries. With the aim of providing consistent estimates of eco-efficiency scores, the study employed a latent-class rather than a conventional DEA model for eco-efficiency analysis. Important heterogeneities in terms of technological choice and AIS characteristics were thus considered when estimating class-specific eco-efficiency scores. Emerging economies, including China, India and Brazil, tend to operate at a different technological frontier than developing country economies, such as Kenya, Uganda and Ethiopia. With exceptions, emerging economies were attributed to class one, while class two predominantly covers developing country economies. These two groups are also fundamentally different in terms of the use of key conventional and environmental pressure variables. As expected, countries in class one (emerging economies) have higher GDP and agricultural output levels. Similarly, the intensity of input use (both conventional and environmental pressure variables) is quite high in these economies compared to countries in class two.

Similarities and differences among classes in terms of the direction and magnitude of the drivers of eco-efficiency are of interest. The quality of the educational system, scientific publishing and the legal right index are positively associated with eco-efficiency levels regardless of class allocation and thus the technological frontier at which countries operate. Similarly, current university-research collaboration in R&D, number of agricultural researchers and ease of accessing loans are negatively associated with eco-efficiency levels regardless of class allocation and thus the technological frontier at which countries operate. However, foreign aid for extension, agricultural policy costs, credit information and spending on health improvements appear to only enhance the eco-efficiency of the countries in class two. According to the results, these countries would benefit more from investments in extension, while countries in class one can boost their eco-efficiency by investing in agricultural research. In general, the results suggest the need for context-specific interventions instead of a “*one size fits all* “approach. While this article illustrates the potential of a macro-level diagnostic approach to assessing the role of innovation systems for sustainability in agriculture, it also demonstrates that care is needed when interpreting results. The evidence generated by this type of analysis can provide potential pointers to policy and investment gaps and opportunities, but inferences should be corroborated with concrete case study data in order to draw sound conclusions.
